# Phase Transitions in Paradigm Shift Models

**DOI:** 10.1371/journal.pone.0070928

**Published:** 2013-08-08

**Authors:** Huiseung Chae, Soon-Hyung Yook, Yup Kim

**Affiliations:** Department of Physics and Research Institute for Basic Sciences, Kyung Hee University, Seoul, Korea; Hungarian Academy of Sciences, Hungary

## Abstract

Two general models for paradigm shifts, deterministic propagation model (DM) and stochastic propagation model (SM), are proposed to describe paradigm shifts and the adoption of new technological levels. By defining the order parameter 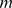 based on the diversity of ideas, 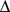, it is studied when and how the phase transition or the disappearance of a dominant paradigm occurs as a cost 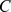 in DM or an innovation probability 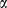 in SM increases. In addition, we also investigate how the propagation processes affect the transition nature. From analytical calculations and numerical simulations 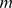 is shown to satisfy the scaling relation 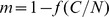 for DM with the number of agents 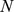. In contrast, 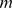 in SM scales as 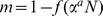.

## Introduction

Transitions are ubiquitous in human history and in scientific activities as well as in physical systems. Human history of civilizations has qualitatively distinguishable periods from stone-age to contemporary civilizations, which depend on dominating themes such as philosophy, art, and technology. In scientific activities such dominating themes correspond to disparate prevailing ideas or concepts such as chaos, complexity, nanophysics, and string theory, which are generally called as *paradigms*. Tomas Kuhn said that the successive transition from one paradigm to another via revolution is the usual developmental pattern of mature science [1]. This paradigm shift is also very similar to the adoption of a new discrete technology level. Examples of such technological levels are operating system versions as Linux distributions and versions of recently-popular smart phones.

To describe the appearance and disappearance of those paradigms, various models [2]–[7] were suggested. In appearance of a paradigm, the propagation of an idea through the social interaction between individuals is essential. To study how the information flow affects the formation of a group sharing common interest in social networks, communication-navigation model with local memory was studied [3]. However this model [3] did not consider the innovation process in which a new paradigm appears and old paradigms disappears. On the other hand, due to its simplicity, two-state interacting spin systems were also widely used to investigate how an existing idea evolves into a dominating theme through the interactions between agents [4]–[7]. Those models were generally focused on the emergence of a single paradigm through social consensus. But, they did not correctly capture the complex dynamical features of paradigm shifts, such as invention of new ideas, competition between ideas, propagation of ideas against the competition to form a new global paradigm, and decline of already existing ones.

Recently, an interesting model was suggested by Bornholdt *et al.* to explain such complex dynamical properties of the paradigm shift [8]. In the Bornholdt model (BM) [8], two essential processes for the paradigm shift were suggested. The two essential processes were never considered simultaneously in previous studies [2]–[7]. In BM each agent 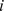 resides on a node of a graph and is assigned an integer 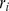. The number plays the role of a particular idea or concept. Then, at any time step the two essential processes are attempted: (i) With probability 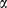 a randomly selected agent 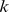 is assigned a new random integer which does not appear anywhere else in the system. Thus 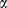 represents the “innovation” rate. (ii) An agent 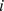 is randomly chosen. Then one of the nearest neighbors 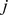 to the agent 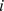 is randomly selected. Denoting by 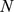 the total number of agents in the system and by 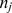 the total number of agents with integer value equal to that of 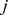, the integer value of the agent 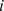 is changed into that of its neighbor 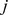 with probability 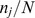, provided that 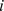 never assumed that particular integer value before. In case it had, then no update is made. The process (i) is the innovation process, in which a new idea or a new paradigm to the whole system is introduced successively. Thus the number of ideas is not limited in BM. The process (ii) is the propagation process. In the propagation process, the memory effect that any agent does not accept any idea experienced before is imposed. This memory effect was also an essential feature of BM. The memory effect was argued to be originated from the part of cultural or scientific activity where people are on an ongoing hunt for new ideas and ideally never return to exactly their old positions [8]. By the numerical study of BM on a square lattice Bornhodlt *et al.* showed the existence of the ordered phase with a globally dominant paradigm for the small innovation probability 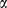 [8]. In this ordered phase the pattern of sudden emergence and slow decline of a dominant paradigm repeats again and again. The epochal things of BM [8] are the innovation process and the memory effect.

Even though Bornhodlt *et al.* showed the existence of a dominant paradigm for small innovation rate 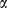, it is still an open fundamental question when and how this ordered phase disappears as 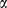 gets larger or approaches to 1. The clear understanding of the transition nature provides more profound physical insight to understand fundamental properties of the system [9]. Furthermore, the propagation of an idea generally occurs successively and continuously or has avalanches as can be seen from the spread of an idea through community networks, social network services and mass communications. Nevertheless the propagation of a paradigm in the process (ii) of BM [8] was only considered to occur locally without avalanche. In addition, the propagation can occur deterministically as the difference (or the gap) of ideas (or technological levels) between two interacting agents grows [10]–[12], whereas the propagation in BM was only considered probabilistically and stochastically.

Therefore, to answer the question when and how the transition occurs from the ordered phase in which a dominant paradigm exists to the disordered phase without any dominant paradigm, and to investigate how the details of propagation process affect the paradigm shifts, we provide two realistic and generalized models for paradigm shifts, deterministic propagation model (DM) and stochastic propagation model (SM). In our models, DM and SM, the innovation process is identical to the process (i) of BM [8]. DM and SM also have the same memory effect as BM. The essential difference between our models and BM is in the details of the propagation process. The details of the propagation are very important in two senses. The first is that the propagation process is the essential mechanism to decide the pattern of sudden emergence and slow decline of a globally dominant paradigm in the system. The second is that the propagation process in a model must reflect the real propagation process in the existing system. The real propagation process should have successive and continuous propagations, i.e., the avalanche. In our models, DM and SM, the propagation process has the avalanche, whereas BM [8] has no avalanche. The real propagation process should also be decided either by the difference of ideas or probabilistically. Therefore we consider two models in this paper. In DM the propagation of an idea between the interacting pair of agents, 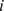 and 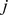, occurs only if the difference of ideas 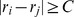. In SM the propagation of an idea between the interacting pair occurs probabilistically and stochastically as in BM.

By defining the order parameter, 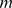, based on the diversity of ideas, 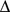, we analytically show that the disappearance of a dominant paradigm can be mapped into the thermal order-disorder transition in physical systems. In DM it is shown that 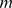 satisfies the scaling relation 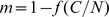. In contrast, 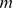 in SM is shown to follow the relation 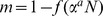, where 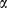 is the innovation probability. Here 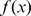 is a scaling function satisfying 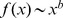 for 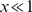 and 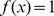 for 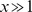. 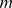 in BM is also proved to satisfy the same scaling relation as 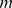 in SM. Therefore, the transition threshold 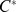 in DM scales as 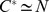, whereas the transition probability 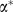 in both SM and BM scales as 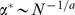. The exponents 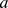 and 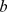 depend both on the propagation mechanism and on the underlying interaction topology of agents. Therefore, from this work, we first provide a standard theoretical framework to understand phase transitions and related phenomena in the paradigm shifts.

### Analysis

To be specific, let’s assume that each agent resides on a node of a certain graph and a pair of nodes connected by a link in the graph is an interacting pair of agents. At a given time 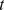 each agent 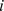 has a non-negative integer 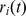, which represents a particular idea or a technological level. In the innovation models of references [10]–[12], the technological level changes continuously or takes rational number. In contrast ideas in this paper take only non-negative integers as in BM [8]. Initially all agents in the system are assumed to have no idea, i.e., 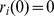 for any 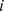. Then at time 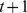, a randomly selected agent 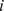 takes an innovation process with the probability 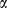 or propagates his idea to other agents with the probability 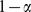. In the process of the paradigm shift, innovation naturally occurs occasionally, whereas propagation occurs frequently and rapidly. Thus, the innovation probability 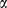 should naturally be very small. In the innovation process at 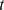, 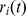 of a randomly-chosen agent 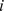 takes a discrete jump to be the smallest positive integer which has not been experienced by any agent in the whole system until the time 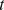 [8]. The propagation process can be a deterministic and rational process or a stochastic and contingent process.

To analyze phase transitions from the ordered phase to the disordered phase of paradigm shift models, we should first understand the model with 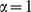, which we call the random innovation model (RIM). Since innovation processes in DM and SM are the same, DM and SM are reduced to RIM at 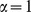. RIM, in which only innovation processes occur without propagation process, cannot have a dominant paradigm any time and is always in the disordered phase. In RIM one can exactly calculate the diversity 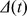, which is defined as 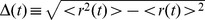, where 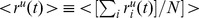 and 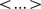 means the average over all possible configurations of 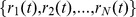. In RIM, a randomly selected agent 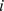 at the time 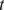 changes his idea into 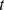 as 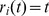. Let’s denote 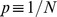 and 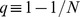, where 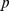 is the selection probability of a particular agent among 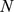 agents. Then the probability 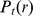 that an agent has the idea 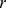 at 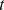 is written as 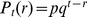 for 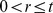 and 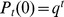. Thus we get
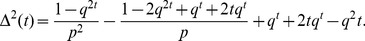
(1)


In the limit 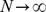,
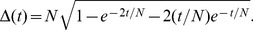
(2)


Equation (2) has been confirmed by numerical simulation. In the steady state (or 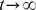), 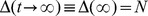. 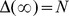 corresponds to the disordered phase for 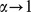 for paradigm shift models. Thus we take the order parameter 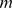 for the phase transition of the paradigm shift models in the steady state as 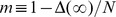. Then 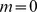 for the disordered phase and 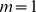 for completely ordered phase with 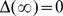, in which all the agents have one same idea. We now consider two different paradigm shift models based on specifics of propagation process.

## Results

### Deterministic Propagation Model

When a new idea (or a new technological level) is created, one normally decides to adopt the new idea by comparing the new idea with his present idea. If the difference between the new idea and the present idea is small, the adoption of the new idea hardly happens. The larger the difference becomes, the more easily one adopts the idea. Therefore the propagation process can depend on the difference in the ideas or the cost [10]–[12]. In this sense, the deterministic propagation model (DM) in which the propagation process is deterministically controlled by the cost is defined in the following way. In the propagation process of DM, a randomly selected agent 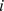 propagates his idea 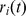 to each nearest neighbor 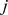, i.e., 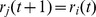 at the time 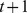, only if 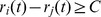. Here 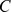 is a constant which represents a propagation cost to adopt a new paradigm. Then the propagation process triggers an avalanche; i.e., if 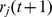 is updated, then repeat the same propagation process for all nearest neighbors of 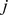. This propagation process is repeated until all the nearest neighbor pairs satisfy the inequality 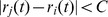.

**Figure 1:**
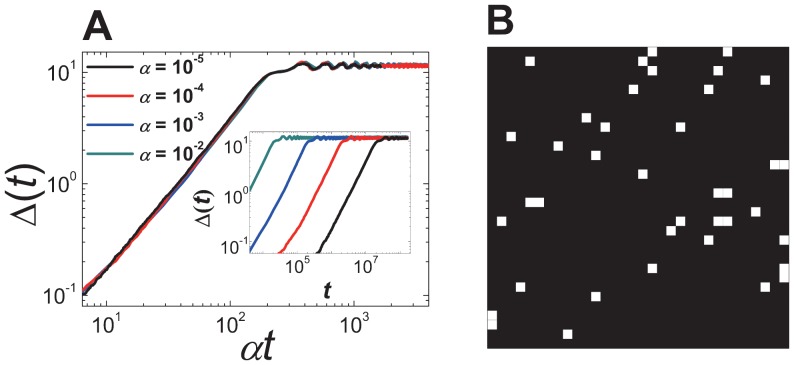
Scaling plot of 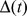 and a snapshot in DM. (**A**) Scaling plot of 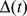 against 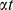 of DM on a square lattice with 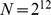 and 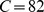. Inset: plot of 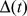 against 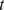. (**B**) A snapshot of a steady state configuration of DM on the square lattice with the size 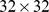. Black dots denote agents with a dominant idea 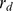. White dots denotes those with ideas different from 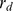.

In DM, 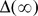 depends only on 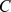 for small 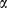 as shown in Figure 1 **A**, because 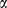 controls only the time 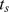 taken for the system to arrive the steady state as 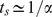. This result physically means that the system is in the steady state if the mean number of innovations, 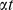, satisfies 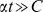 and the physical properties of the steady state depend only on 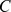.

First we consider DM on the complete graph (CG). Each agent on CG is a nearest neighbor of all the other agents. Therefore one propagation process from a randomly-selected node makes propagation tries to all the other agents. Let’s think a steady state configuration that ideas in the system spread in an integer set 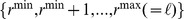 when the 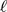-th innovation process happens. In the average sense 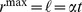 if the 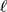-th innovation happens at 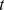. If 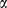 is small enough, there should exist a propagation process initiated from an agent with 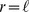 among the many propagation processes before the 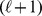-th innovation process occurs. The propagation process from the agent with 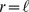 makes the configuration with 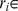 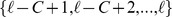. Then, considering the fact that the 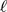-th innovation drives the 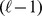-th configuration with 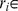 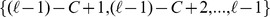 into the 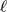-th configuration with 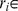 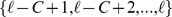, the probability 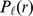 that an agent has an idea 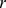 in the 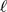-th configuration satisfies recursion relations
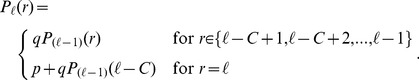
(3)


By applying the recursion relations (3) 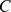 times, we obtain
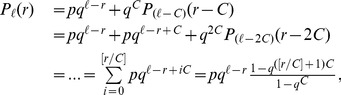
(4)where 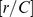 is an maximal integer which is not greater than 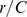. In the limit 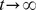 or 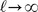, 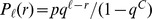 and 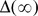 is written as



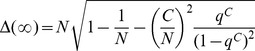
(5)In the large 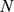 limit, 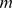 thus satisfies
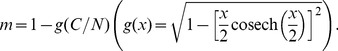
(6)


**Figure 2:**
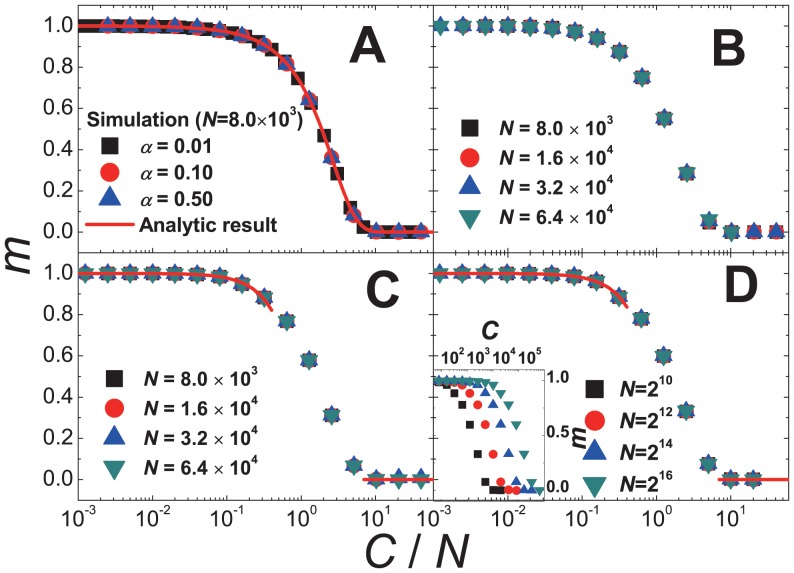
Analytic and simulation results of DM. Scaling plots of 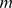 against 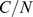 of DM (**A**) on the complete graph with 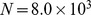, (**B**) on a scale-free network (**C**) on a random network and (**D**) on a square lattice. Curves in the figures show the analytic results Equation (6) and Equation (9). All the simulation data in **B**, **C** and **D** are obtained by use of 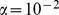. Inset of **D** shows the plots of 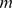 for various 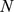 against 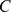.

Even though Equation (6) was derived under the physical assumption that 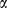 is very small, Equation (6) agrees very well with the simulation results for quite large 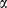 or for 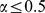 as shown in Figure 2 **A**. The ordered state of DM on CG has a peculiar physical property. Since 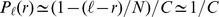 for 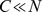, there doesn’t exist a unique dominant idea, but 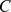 ideas are nearly equally probable in the steady state. This peculiar ordered state comes from the global connectivity of CG. In the sense that DM naturally regards ideas within the difference 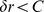 as the same one, the ordered state on CG is physically plausible and understandable.

In contrast, there exists a unique dominating idea in DM on other graphs with local connectivity for 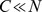 as shown in Figure 1 **B**, Figure 2 **B**, **C** and **D**. Thus we now want to analytically show the existence of the ordered state with a dominating idea on the graphs with local connectivity. In DM any nearest neighbor pair 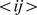 of agents should satisfy the condition 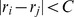 after a propagation process. Let’s first think about the configuration with the 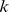-th dominating macroscopic idea 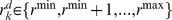. Now we want to show how the configuration with the 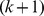-th dominating idea 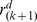 happens. As shown in Figure 1 **B**, the nodes (or sites) with 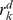 form a macroscopic percolation cluster through the links (or bonds) of the graph and the nodes with 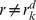 form only isolated microscopic clusters. Thus the propagation process which changes the dominating idea happens only through the macroscopic percolation cluster. Therefore the configuration with the 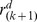 does not happen until the idea 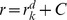 appears in the system. After the idea 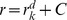 appears, subsequent propagation processes through the macroscopic cluster which make the configuration with 
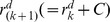
 appear before the next innovation process happens. The configurations with 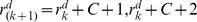 and … are also possible, but the probabilities that these exceptional configurations happen are nearly negligible if 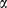 is small and 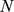 is large. So we neglect these exceptional configurations in the subsequent calculations. In the configuration with 
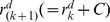
, the ideas in the system spread in the set 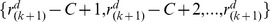. Then before the configuration with 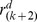 appears, the configuration of the system can evolve into one in which the ideas spread in the set 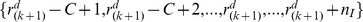 with 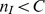. Here 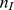 is the number of the innovations which occur before the configuration with 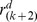 appears. Generally the system in the steady state has a configuration with the ideas spread in the set 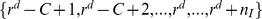.

Now we consider the probability 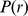 that an agent has an idea 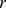 in the steady state. Clearly 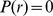 for 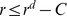 and 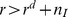. Furthermore, in the large 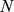 limit 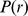 is expected to satisfy 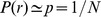 for 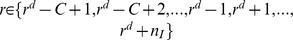, because an idea in this set is originated from an innovation process. From the continuum limit 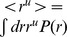, we get 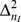 as
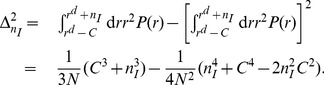
(7)


Since 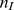 is equally probable to be any integer in the set 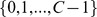,
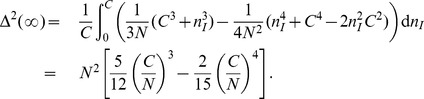
(8)


Therefore, 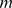 for 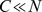 satisfies 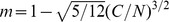. For 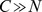, DM reduces to RIM and 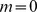. Thus 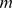 satisfies the scaling relation
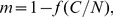
(9)where 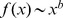 with 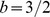 for 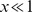 and 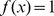 for 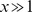. On CG the same scaling relation with 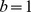 holds for 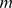.

To confirm the scaling relation (9) on the graphs with local connectivity, DM is studied by simulations on various graphs. The graphs used in this paper are a scale-free network with the degree exponent 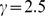 [13], and an Erdös-Rényi type random network, and a two-dimensional square lattice. To accord with the square lattice, the mean degree 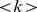 of the scale-free and random networks is set as 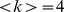. The simulation data of 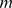 on each graph in Figure 2 are obtained by averaging over at least 1000 realizations. The scaling relation of 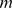 with 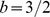 or Equation (9) is confirmed by simulations on the random network and the square lattice as shown in Figures 2 **C** and **D**. In contrast, on a scale-free network with degree exponent 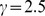, the scaling relation with 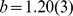 is obtained (Figure 2 **B**). The deviation of the exponent 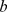 from 3/2 on the scale-free network is probably explained from the hub effect of the scale-free networks with 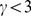, which provides an aspect of global connectivity. Thus 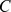 at which the phase transition occurs, 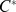, scales as 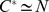 on arbitrary graph.

Even though the scaling relation (9) was derived under the physical assumption that 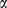 is very small, we have confirmed that Equation (9) agrees very well with simulation results for quite large 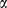 or for 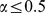 on the square lattice and the random network as on CG (Figure 2 **A**).

### Stochastic Propagation Model

**Figure 3:**
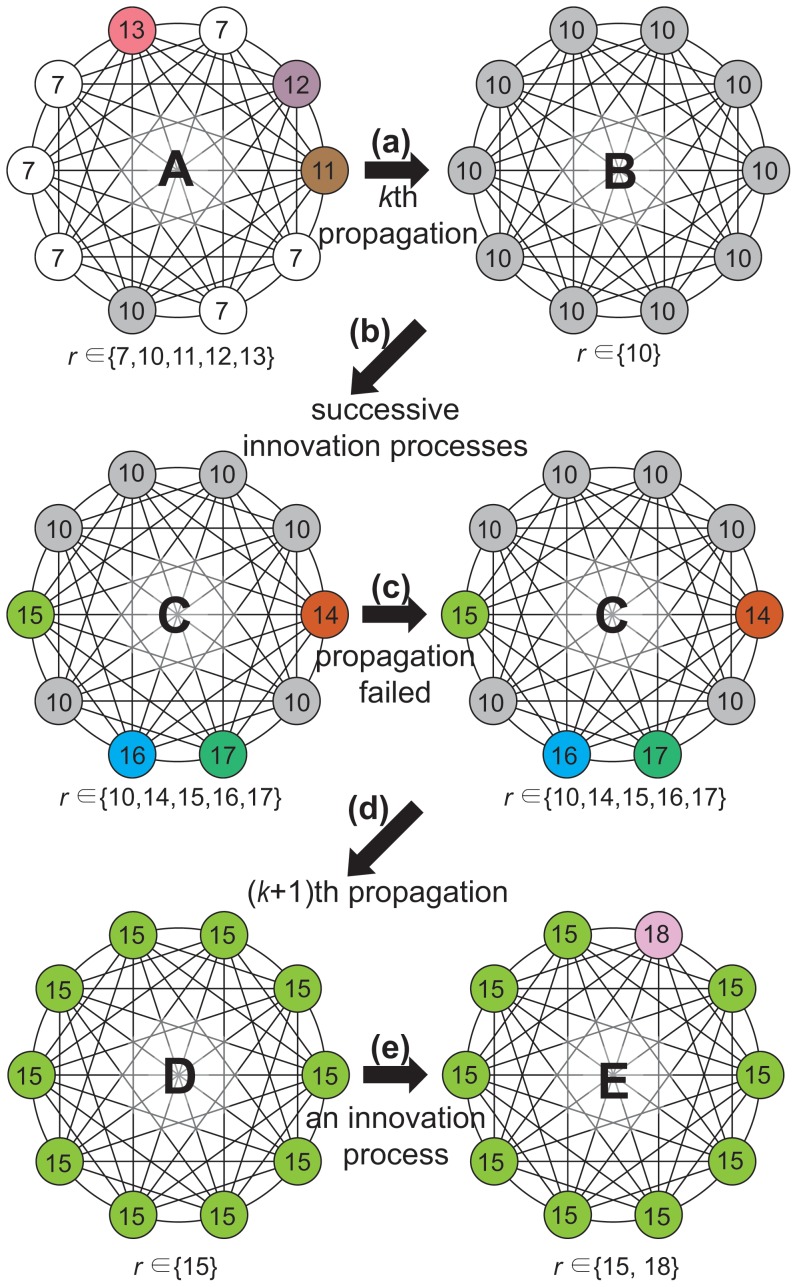
Schematic diagram for the evolution of configurations in SM on CG. **A** is a configuration with 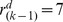 and 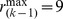. The next propagation process (a), (or the 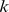-th propagation) at 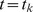, changes **A** into **B** with all 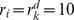 and 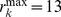. Successive innovation processes (b) change **B** into **C** with 
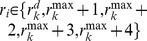
 with 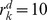 and 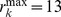. The propagation process (c), which cannot be executed by the probability 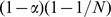, leaves **C** as it is. The propagation process (d) at 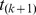 initiated from an agent with 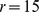 drives **C** into **D** with all 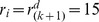 and 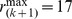. An innovation process (e) drives **D** into **E** with 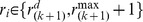 (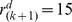 and 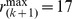).

We now consider the stochastic propagation model (SM) in which the propagation process occurs probabilistically and stochastically. In SM, the feature that a minority idea is more difficult to be adopted than a more widespread idea [8] is considered. Therefore, the propagation process in SM is defined in the following way. If a propagation try is taken at a given time 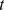 with the probability 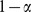, first a site 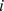 with the idea 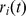 is randomly selected. Then with the probability 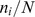 the propagation process starting from the site 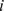 occurs. Here 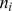 is the number of agents in the system which have the same idea with 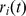. If the propagation process happens, then the ideas of all nearest neighbors of 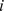 are simultaneously made to be equal to 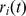, except the ideas of neighbors who have experienced 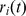 before. In addition, all the neighbors whose ideas are changed also propagate the idea 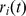 to all of their nearest neighbors in the same manner with the updated probability 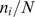, because 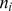 increases as propagations continue. The propagations continue until the propagations are terminated by the probability 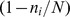 or all the agents in the system are tried to be propagated. Therefore, the propagation process of SM also has the avalanche and an idea 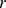 can spread to the whole system for one time step. Moreover, as we shall see, the scaling properties of SM on graphs with local connectivity are the same as those of BM [8].


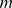
 of SM on CG is analytically calculable, because an idea propagates to the whole system by single propagation process. For the calculation one should understand the time evolution of configurations in SM on CG. The schematic diagram for the evolution is shown in Figure 3. For the explanation of the evolution, let’s define the maximal 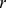, 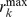, appeared in the system until the time 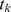 at which the 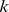-th dominant idea 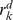 appears.

A typical configuration at 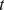
(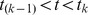) is one with 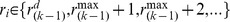 (see Figure 3 **A**). Then the next propagation process drives this configuration into one with all 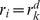, where 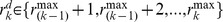 (see the process (a) in Figure 3). Then successive innovation processes make the configuration with 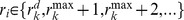 (see the process (b) in Figure 3). Note that the propagation process which cannot be executed by the probability 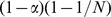 does not change the configuration of system (see the process (c) in Figure 3 ). Then the 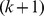-th propagation process drives this configuration into one with all 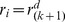 with 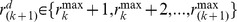 (see the process (d) in Figure 3). Then an innovation process drives the configuration with 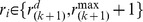 (see the process (e) in Figure 3). In the steady state of SM, this evolution pattern is repeated again and again. Thus now we analytically calculate 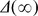 or 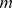 of SM based on this evolution pattern.

Now we consider the probability 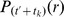 that an agent has the idea 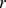 at 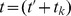 with 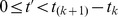. In the average sense, the number of innovations occurring from 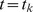 to 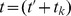 is 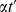. Then at 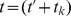, similar to RIM, 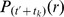 is written as
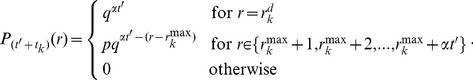
(10)


**Figure 4:**
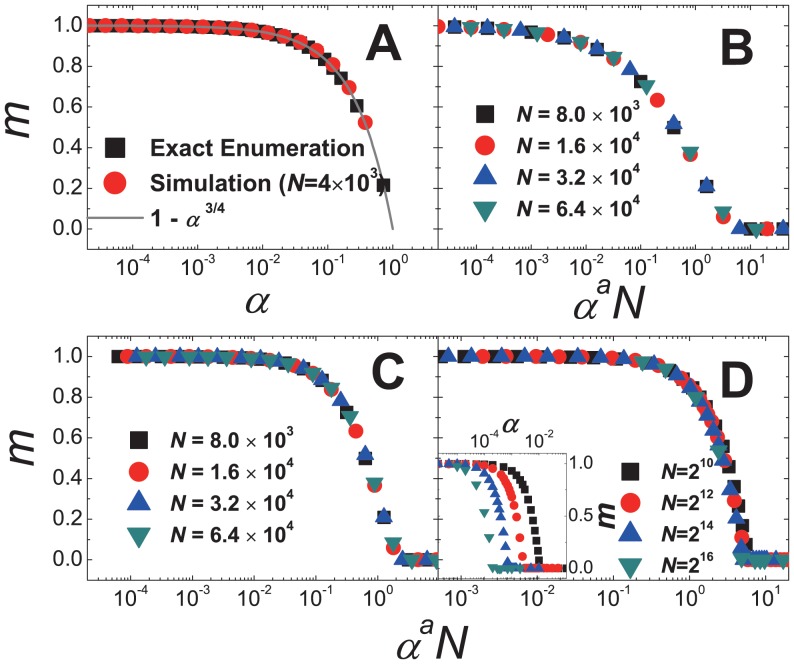
Analytic and simulation results of SM. (**A**) Plot of 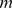 against 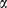 of SM on the complete graph. The data both from the exact enumerations and simulations are shown. The curve represents the analytic result 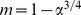. (**B**-**D**) Scaling plots of the simulation data for 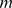 of SM against 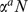 on the scale-free network (**B**), on the random network (**C**) and on the square lattice (**D**). Inset of **D** shows the plots of 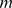 for various 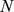 against 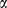.

Thus from 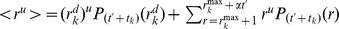, we get 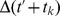 of the configuration with 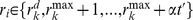 for large 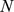 as
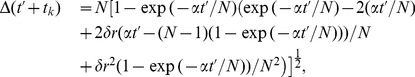
(11)with 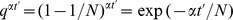 and 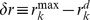. 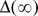 is thus written as
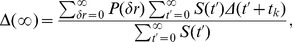
(12)where 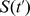 is the probability that no propagation processes happen from 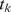 until 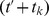 and 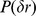 with 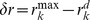 is the probability that a configuration with 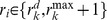 occurs at the very next innovation process after 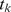 or at 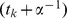 in the average sense (see Figure 3 E). Now we calculate 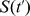. The probability that a propagation process at 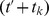 can be executed is 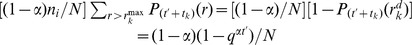 with 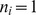 and 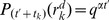. Then 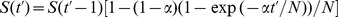 in the large 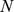 limit. By taking the continuum time limit,



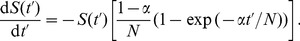
(13)Thus we get
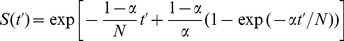
(14)from 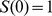. Now we want to calculate 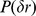. At 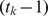, 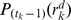 that an agent has the idea 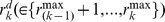 is 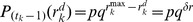. Then the propagation process to make 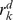 the 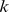-th dominant idea occurs with the probability 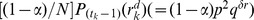. Since the probability for 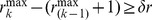 is 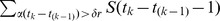, 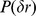 can be written as



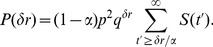
(15)From Equations (11), (12), (14) and (15), 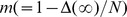 on CG can be calculated through exact enumeration. The results of the exact enumerations are shown in Figure 4 **A**.

For small innovation probability 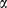, 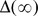 or 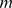 can be analytically calculable. For 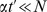, 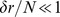. Thus
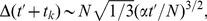
(16)

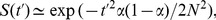
(17)and
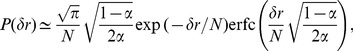
(18)where 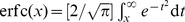. Therefore, from 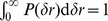 we get 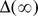 as
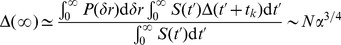
(19)for 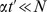, and 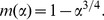
(20)We also confirm that Equation (20) holds even for quite large 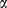 or for 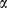 close to 1 by comparing Equation (20) with the results of the exact enumerations as shown in Figure 4 **A**. This result means that there always exists a dominating idea or the global paradigm on CG if 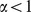.

On the graphs only with local connectivity, the analytic approach as on CG to SM is hardly possible. Instead simulations are carried out. The simulation results on various graphs with local connectivity show that 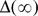 satisfies the scaling ansatz 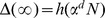 very well. As shown in Figure 4, 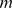 satisfies the scaling function similar to that of DM as
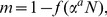
(21)where 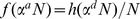. {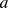, 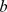} are {2.01(3), 0.49(2)} on the scale-free network, {1.15(2), 1.05(3)} on the random network, {1.10(2), 1.13(2)} on the square lattice. Thus the phase transition probability 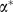 scales as 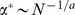 and 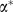 decreases as the global connectivity of the graph decreases. Moreover the exponent 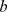 increases as the global connectivity decreases. The scaling behavior of SM on the random network is nearly equal to that on the square lattice. This result means that the scaling behavior hardly depends on the dimensionality of the graph, but depends on the connectivity.

We also study 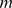 of BM [8]. In BM, a randomly selected agent 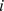 tries to propagate his idea to a randomly chosen nearest neighbor with the probability 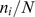. No further propagation process is attempted in BM or BM does not allow the avalanche in a propagation process. Since the propagation in BM is local, it is difficult to treat the model analytically even on CG. Thus BM is studied numerically. From the simulations we confirm the same scaling behavior 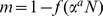 with 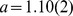 and 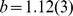 on any graph, especially on CG. The scaling behavior of BM on any graph is the same as those of SM on the square lattice. BM has only local propagation process on any graph and does not use the connectivity of large scale or the global connectivity, even on CG. Therefore the scaling properties of BM are irrelevant to the dimensionality or the connectivity of the graph. SM on the square lattice physically has only local avalanches, and thus the scaling properties of SM on the square lattice are the same as those of BM. 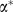 of BM also scales as 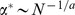 with 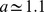.

## Conclusion and Discussion

We introduce two paradigm shift mechanisms as the deterministic propagation model (DM) and the stochastic propagation model (SM). Both models have the memory effect that an agent never returns to any paradigm experienced before by any process as BM. In both models there commonly exists the innovation process, which occurs with the probability 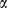. With 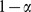 the propagation process occurs. Both DM and SM have the avalanche in the propagation process. In DM, the propagation process is controlled by the cost 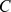, which represents the idea difference or resistance to make one adopt a new idea. In contrast, the propagation process of SM occurs probabilistically and stochastically by considering the feature that that a minority idea has more difficulty for adoption than a more widespread idea.

To analyze phase transitions from the ordered phase with a dominant paradigm to the disordered phase in paradigm shift models, the disordered phase is exactly defined by using the random innovation model (RIM) in which the diversity of ideas 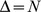. By defining the order parameter, 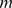 as 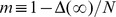, we first provide a novel theoretical framework in which transition in paradigm shift models is analyzed quantitatively by applying the scaling theory of statistical physics for the analysis of the traditional thermal order-disorder transition. In DM 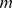 of the steady state satisfies the scaling relation 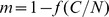 on any graphs. In contrast, 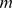 in SM follows the scaling relation 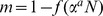. Here 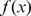 is a common scaling function satisfying 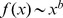 for 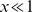 and 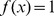 for 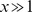. 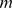 of BM [8] on any graph is also proved to satisfy the same scaling relation as 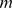 of SM on the square lattice. Thus, in DM the transition threshold 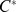 scales as 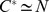 and the transition probability in both SM and BM scales as 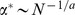. The exponents 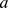 and 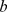 depend both on the models and on the underlying interaction topologies.

Thus this paper suggests a novel theoretical method based on the scaling theory of the statistical physics to understand the phase transitions in social systems such as paradigm shifts quantitatively. The resultant scaling relations in DM and SM also quantitatively and exactly show that there cannot exist a dominant paradigm if innovations happen too frequently or the resistance to make one adopt a new idea becomes large in the systems with finite 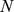.

The deterministic and stochastic propagations coexist in real world. Thus, it would be an interesting open question how the nature of the phase transition and the dynamical properties in paradigm shifts are affected by the coexistence of two processes. Furthermore, it would also be very interesting to apply the paradigm shift models (DM, SM, and BM) to the analysis of the real data for the paradigm shifts or the technological level shifts. One of the such real data should be the adoption patterns of operating system versions or versions of recently-popular smart phones. Another interesting future study would be to investigate modified versions of the innovation models in which the technological level changes continuously or takes rational number [10]–[12]. Such innovation models [10]–[12] only considers the deterministic propagation process. As emphasized previously, any propagation should have both stochastic and deterministic aspects. Therefore it would also be very interesting to investigate the innovation model with continuously varying technological levels and the combination of deterministic and stochastic propagation processes.
